# Alleviating Nurse Burnout With an Artificial Intelligence–Selected Mobile Cognitive Behavioral Therapy–Based Intervention: Mixed Methods Randomized Controlled Trial

**DOI:** 10.2196/85986

**Published:** 2026-07-03

**Authors:** Yeongeun Kim, Chiyoung Cha, Gumhee Baek

**Affiliations:** 1 Dongnam Institute of Radiological and Medical Sciences, Busan, Republic of Korea; 2 College of Nursing, Ewha Womans University, Helen Hall 112, 52, Ewhayeodae-gil, Seodaemun-gu, Seoul, 03760, Republic of Korea, +82 02-3277-2876; 3 Ewha Research Institute of Nursing Science, Ewha Womans University, Seoul, Republic of Korea

**Keywords:** artificial intelligence, burnout, cognitive behavioral therapy, mHealth, mobile health, nurse, randomized controlled trial

## Abstract

**Background:**

Nurse burnout is a pervasive global problem. Cognitive behavioral therapy (CBT) has been shown to reduce burnout; however, most digital CBT programs use standardized approaches that overlook individual differences in burnout profiles. With advances in artificial intelligence (AI), algorithm-based recommendation systems now enable personalized intervention delivery by matching specific CBT modules to users.

**Objective:**

This study aimed to test the effects of an AI-selected mobile CBT-based intervention on nurse burnout and to describe participants’ experiences with the intervention. Specifically, it evaluated whether an AI-selected CBT-based intervention differentially reduced burnout subdomains compared with an information-only control group and explored how nurses perceived and engaged with the AI-selected program.

**Methods:**

This study adopted a mixed methods design, integrating a 2-group randomized controlled trial and qualitative content analysis exploring participants’ experiences. For this randomized controlled trial, a total of 125 nurses were enrolled and randomly assigned to either the experimental group (n=62) or the control group (n=63) between October 2024 and December 2024. The experimental group received an AI-selected mobile CBT-based intervention, in which an AI algorithm assigned CBT modules based on participants’ burnout profiles (client-related, personal, and work-related), job stress, and coping characteristics. The control group received information related to burnout management. Primary outcomes, client-related, personal, and work-related burnout, were assessed at baseline, 2 weeks, and 4 weeks. Secondary outcomes, including coping strategies, job stress, and stress response, were assessed at baseline and 4 weeks. Between-group differences in burnout over time were examined using repeated measures analysis of variance, with adjustment for job stress and stress response. Within-group changes and postintervention group differences were analyzed using *t* tests. Open-ended survey responses and follow-up interviews (n=5 in the experimental group) were analyzed using thematic content analysis.

**Results:**

Follow-up completion rates were 84.6% (137/162) at both 2 and 4 weeks. The experimental group showed a greater reduction in client-related (*F*_1,121_=7.548; *P*=.007), personal (*F*_1,121_=6.533; *P*=.01), and work-related burnout (*F*_1,121_=38.194; *P*<.001) than the control group, reflecting more pronounced within-group improvements over time. No significant between-group differences were observed for coping strategies, job stress, or stress response. Qualitative findings suggested that some participants were receptive to the AI-selected CBT-based intervention and reported increased self-awareness and reflective engagement with coping strategies that they might not have selected independently.

**Conclusions:**

The findings suggest that participants were receptive to AI-selected CBT-based interventions, suggesting the potential of such interventions as a supportive approach for alleviating nurse burnout. Future research should explore the sustainability of these effects and optimize the intervention duration to enhance engagement and impact.

## Introduction

Nurses play an essential role in delivering health care services and represent nearly half of the global human resources in the health sector [1]. However, many nurses are increasingly vulnerable to burnout due to prolonged exposure to physical and psychological stressors in clinical settings [2]. Several meta-analyses have estimated that the prevalence of burnout among nurses ranges from 11% to 56% [3-5], and in Korea, 55.9% of frontline nurses experience burnout [6].

Nurse burnout is multifaceted and often categorized into 3 subdomains: client-related burnout (exhaustion related to patient care), personal burnout (emotional and physical exhaustion), and work-related burnout (stress stemming from job demands) [7]. Given the ongoing nursing shortage [8] and rising health care demands [9], accessible and effective interventions are required to address burnout. These interventions should consider not only general experiences of burnout but also its distinct subdomains, which may vary according to individual roles and work contexts.

Cognitive behavioral therapy (CBT) is a structured, problem-focused intervention that aims to modify maladaptive and irrational cognitions underlying emotional and behavioral issues [10]. Beyond traditional protocol-based CBT, an expanding body of research has applied CBT-based or CBT-informed psychological interventions, including mindfulness-based approaches [11], laughter therapy [12], storytelling [13], and reflective writing [14], to address burnout-related symptoms. CBT has been widely recognized as an effective intervention for reducing burnout symptoms among nurses by helping them restructure their dysfunctional thoughts and behaviors across various clinical settings. Meta-analytic evidence suggests that CBT is particularly effective in alleviating emotional exhaustion [15] and depersonalization [16], which are 2 core subdomains of burnout. These findings highlight the potential of CBT-based interventions to effectively address key aspects of nurse burnout.

With the rapid advancement of mobile technology, mobile health (mHealth) interventions have become increasingly common in supporting mental health [17]. These platforms offer scalable, flexible, and cost-effective alternatives to traditional face-to-face therapy, making them particularly useful for health care professionals, such as nurses, who often face temporal and spatial barriers to accessing psychological support [18]. Application-based CBT programs, such as Wysa [19] and Woebot [20], have shown effectiveness in reducing symptoms of depression and stress. Artificial intelligence (AI)–based recommendation systems are increasingly being integrated into mHealth platforms to enable the delivery of personalized interventions [21]. Originally developed and widely adopted in industries such as retail and entertainment, collaborative filtering and content-based recommendation algorithms are currently being utilized in health care settings to tailor interventions based on user profiles, symptom severity, and behavioral patterns [22]. These intelligent systems have the potential to enhance user engagement and therapeutic outcomes by offering interventions that optimally match individual needs [23].

Despite growing evidence supporting the effectiveness of CBT-based interventions in reducing burnout among nurses, most studies have focused on evaluating the effects of a single standardized program [16]. These approaches often adopt a one-size-fits-all model, overlooking the multidimensional nature of burnout and the diverse needs of individual users. Few interventions have addressed burnout subdomains as distinct targets for tailored support. Moreover, there is a notable lack of research that applies user-based recommendation systems to suggest interventions based on nurses’ individual characteristics, such as their burnout profiles or professional backgrounds. This gap highlights the need for an adaptive and tailored system that can enhance the effectiveness and relevance of digital mental health interventions in the nursing workforce.

This study evaluated the effectiveness of an AI-selected mobile CBT-based intervention designed to reduce burnout among nurses. The intervention used a user-based recommendation system to enhance its impact by suggesting 1 of 4 CBT-based modules tailored to the user’s burnout subdomain, as well as demographic and work-related factors. Previously, we conducted a 3-group randomized trial using this system [24], which demonstrated significant improvements in some burnout subdomains.

Since then, the algorithm has been further optimized, but it remains unclear how participants perceive and engage with programs selected by AI. To address both issues, we designed this study to evaluate the intervention using quantitative and qualitative approaches, thereby providing a more comprehensive understanding of its effectiveness and user acceptance.

## Methods

### Study Design

We adopted a mixed methods design, integrating a 2-group randomized controlled trial (single-anonymized) and qualitative content analysis exploring participants’ experiences. The study was conducted in accordance with the CONSORT (Consolidated Standards of Reporting Trials) guidelines for reporting randomized controlled trials [25] (Checklist 1). In addition, the qualitative component of this study was reported in accordance with the COREQ (Consolidated Criteria for Reporting Qualitative Research) checklist [26].

### Randomization

Randomization was conducted using an online tool (randomizer.org, a service provided by the Social Psychology Network), ensuring an equal 1:1 allocation between the experimental and control groups. Participants were not informed about the randomization before enrollment to reduce selection bias and preserve objectivity during the intervention process.

### Participants and Sample Size

Participants were recruited through 2 large South Korean online nurse communities (1500 registered nurse-verified members). Recruitment notices containing the study purpose, procedures, and inclusion criteria were posted as online announcements. Nurses who wished to participate accessed a Google Forms survey link, reviewed the study information, and provided electronic informed consent. Inclusion criteria were nurses who had completed preceptorship and worked independently for at least 1 month; those not providing direct care or with prior exposure to mobile burnout interventions were excluded. Additionally, participants were allowed to withdraw from the study after enrollment if (1) personal- or health-related circumstances made continued participation impossible, (2) they chose not to complete the study, or (3) they had difficulty participating in the study due to safety or ethical reasons.

We estimated the required sample size using G*Power (version 3.1.9.7) [27], based on a previous study [28], with an effect size of 0.13, α=.05, power=0.95, and 3 repeated measures, resulting in 130 participants. Allowing for a 20% attrition rate [29], the recruitment target was set at 162 participants (81 per group). To ensure data quality, baseline variables were screened for univariate outliers. Observations more than 1.5 to 3 IQRs from the median were removed, resulting in the exclusion of 12 cases (experimental=4; control=8). Of the 162 eligible participants randomized, 137 (84.6%) completed both the 2-week (posttest 1) and 4-week (posttest 2) follow-up assessments. For the final quantitative analyses, 125 participants (experimental=62, control=63) were included after the data-cleaning process for missing values and outliers, indicating a 77.2% retention rate for a fully remote intervention.

### Interventions

#### Experimental Group

The intervention designed by the research team was delivered via a mobile app called Nurse Healing Space. This app comprises 4 distinct modules that target burnout reduction: (1) mindfulness meditation, (2) storytelling and reflective writing, (3) laughter therapy, and (4) acceptance and commitment therapy (Table 1). The experimental group received AI-selected CBT-based intervention modules at 2-week intervals. Over the 4-week intervention period, the AI algorithm sequentially selected 2 modules from the 4 available CBT-based interventions. Participants engaged in 3 sessions per week, with each session lasting approximately 10 to 15 minutes. Animated female characters appeared in the videos to guide participants through the activities. For the mindfulness meditation, users followed guided instructions regarding body movements, breathing techniques, and meditative practices. Storytelling and reflective writing involved watching narratives shared by fellow nurses, followed by reflective writing about their personal experiences. In laughter therapy, participants were led through songs and dance routines designed to elicit positive affect and laughter. The acceptance and commitment therapy component focused on emotional acceptance and thought regulation, as modeled in the videos. All program content was developed with input from domain experts to ensure both clinical relevance and psychological effectiveness.

**Table 1.** Program components of intervention.

Week and session	Programs			
	Mindfulness meditation	Storytelling and reflective writing	Laughter therapy	Acceptance and commitment therapy
Week 1				
Session 1	Body scan (upper body), self-compassion meditation	Work is piling up	Look like my mom, bee dance	Hello my heart (introduction)
Session 2	Body scan (lower body), yoga meditation	I am a person too	Don’t make a wry face, bee dance	Thinking is just thinking (cognitive diffusion)
Session 3	Sitting up straight meditation, relaxation meditation	There aren’t enough hours in the day	When I go to Los Angeles, creative movement	This is the moment (being present in the moment)
Week 2				
Session 4	Body scan (upper body), yoga meditation	Am I an emotional outlet?	Look like my mom, open chest 1, 2, 3	Look back at me (self as context)
Session 5	Body scan (lower body), sitting up straight meditation	The working environment is too poor	Don’t make a wry face, free movement	Worthy life (the best 3 moments of my life)
Session 6	Self-compassion meditation, relaxation meditation	There is a shortage of nurses	When I go to Los Angeles, creative movement	Let’s do it together (committed action and willingness)

The algorithm was developed to provide personalized burnout interventions by identifying the most similar prior participant using both profile similarity scores and burnout patterns. The development, operation, optimization, and intervention content of the AI algorithm are discussed in greater detail in a separate study [30].

After completing Program 1, participants’ burnout levels were reassessed. If the most elevated burnout dimension showed a reduction of 50 points or more, the data were considered valid and incorporated into the algorithm. Otherwise, the data were considered invalid and excluded from AI model training, although the participants continued to receive the subsequent intervention modules without restriction. As data accumulated, the AI continually updated its artificial neural network through iterative learning. Based on the reassessed burnout dimension scores after Program 1, the AI algorithm recommended a second 2-week program (Program 2) using the same algorithm. The variables were reassessed after Program 2. Following this, the AI algorithm updated its artificial neural network upon recognizing valid data. Participants in the experimental group completed a pretest survey (107 items: demographic and work-related characteristics and study variables), followed by Program 1 for 2 weeks, posttest 1 (19 items: burnout), Program 2 for 2 weeks, and posttest 2 (97 items: study variables).

#### Control Group

Participants in the control group were provided with online informational materials covering all 4 intervention programs (mindfulness meditation, storytelling and reflective writing, laughter therapy, and acceptance and commitment therapy) through a blog platform. We sent the URL to participants after they had completed the pretest survey. The control group also completed the same sequence of assessments as the experimental group: a pretest survey, posttest 1 at week 2, and posttest 2 at week 4. After completing the final assessment, participants in the control group were given access to *Nurse Healing Space*.

### Measurement

#### Demographic and Work-Related Characteristics

Data included age, sex, marital status, job title, hospital size, clinical experience, department, overtime, shift type, and turnover intention (0‐10 scale).

#### Primary Outcome

We assessed burnout using the Korean-adapted Copenhagen Burnout Inventory [7,31]. This 19-item instrument measures client-related, personal, and work-related burnout on a 5-point Likert scale. Each item is transformed to a 0 to 100 score, and subscale scores are calculated as the mean of the corresponding items, with higher scores indicating greater burnout. The possible score range for each subscale is 0 to 100. Cronbach α in this study was 0.936.

#### Secondary Outcomes

We measured coping using the Korean version of the Coping Strategy Indicator [32,33]. The 33-item scale includes social support-seeking, problem-focused, and avoidance-focused coping, rated on a 3-point Likert scale. Subscale scores are calculated by summing the corresponding item scores (possible range: 11‐33), with higher scores indicating greater use of each coping strategy. Cronbach α in this study was 0.888. We measured job stress with a 23-item scale developed for Korean nurses [34,35]. It covers 6 subdomains: workload overload, role conflict as a professional, lack of professional knowledge and skills, interpersonal relationship problems, inappropriate treatment and compensation, and night shift. These are rated on a 5-point Likert scale. Item scores range from 1 to 5, and the overall job stress score is calculated as the mean of the item scores, with higher scores indicating greater job stress. The possible score range is 1 to 5. Cronbach α in this study was 0.881. We measured stress response with the 22-item Stress Response Inventory-Modified Form [36,37], covering somatization, depression, and anger, rated on a 5-point Likert scale (0‐4). Total scores range from 0 to 88, with higher scores indicating greater stress response. Cronbach α in this study was 0.936.

### Data Collection

Data were collected between October 8 and December 31, 2024. Quantitative data were assessed at baseline, 2 weeks, and 4 weeks. Participants advanced to the next program only after completing the previous one. Although each module was designed to last 2 weeks, actual usage varied: Program 1 ranged from 14 to 20 days (mean 15.80, SD 1.30 d) and Program 2 ranged from 14 to 18 days (mean 15.55, SD 1.0 d). The integrated management platform enabled the research team to monitor participants’ study progress and completion status at each stage of the study. To support continued participation, weekly reminder messages were sent during the intervention period, and participants received compensation after completion of each survey assessment point.

Qualitative data were collected to explore participants’ experiences with the AI-recommended interventions. As part of the survey, all experimental group participants completed a short open-ended questionnaire. Following the completion of the intervention, participants in the experimental group were contacted via text messages to assess their willingness to participate in one-on-one follow-up Zoom interviews, and 5 agreed to participate. Each interview lasted approximately 30 minutes (Textbox 1). The semistructured interview guide was collaboratively developed by the research team and reviewed by an experienced qualitative researcher. The follow-up interviews were conducted by the first author, a female clinical nurse and doctoral student with approximately 10 years of inpatient experience and prior qualitative research experience. No prior relationship was established between the interviewer and participants before the study, and participants were informed of the interviewer’s role as a researcher and the purpose of the study prior to participation. To minimize potential interviewer bias, the interviewer maintained reflexive memos during data collection, adhered to the semistructured interview guide, and engaged in peer debriefing with an experienced qualitative researcher. These procedures helped enhance the credibility and trustworthiness of the qualitative findings. With participants’ consent, all Zoom interviews were audio-recorded for transcription and analysis purposes. No video recordings were made, and participants were informed that they could freely disable their cameras during the interview.

Textbox 1. **Interview questions.**

1. If you were to describe the program you completed to a friend, what would you say?
2. In what ways did the program help reduce your burnout?
3. What positive experiences did you have while participating in the recommended program?
4. What negative experiences did you have while participating in the recommended program?
5. What was the most significant change you noticed before and after completing the program?
6. What are your thoughts on the use of artificial intelligence (AI) in health programs?
7. During the intervention period, were you aware that the program you received had been selected by an AI algorithm?
8. How do you feel about receiving and following a program recommended by an AI algorithm?
9. How do you think your experience would differ if you chose a program yourself compared with following one recommended by an AI algorithm?

### Data Analysis

We analyzed data using SPSS Statistics (version 27; IBM Corp). Descriptive statistics summarized participants’ characteristics and study variables. We tested baseline homogeneity between groups using chi-square tests and *t* tests. Group differences in burnout over time were examined using repeated-measures ANOVA, with job stress and stress response as covariates due to their significant correlations with burnout. We assessed within-group changes in coping strategies, job stress, and stress response using independent *t* tests. Within-group changes in burnout from baseline to posttest 2 were further examined using paired-sample *t* tests, and effect sizes were calculated using Cohen *dz*. An intention-to-treat (ITT) analysis was not conducted. This study used a sequential AI-based intervention in which outcome data at each assessment point were required to inform subsequent algorithm-selected module recommendations and to ensure the validity of adaptive intervention delivery. Accordingly, the primary quantitative analyses were conducted using participants with complete outcome data across assessment points. The absence of an ITT analysis is acknowledged as a methodological limitation of this study.

Qualitative data were analyzed using content analysis to identify participants’ experiences with the AI-based CBT intervention. For this qualitative component, an inductive content analysis approach was used. The first author repeatedly read the interview transcripts and open-ended responses to become familiar with the data and generated initial open codes. These codes were then organized into broader categories through an iterative comparison process. To enhance the credibility of the findings, a peer debriefing procedure was conducted, in which an experienced qualitative researcher reviewed the coding structure and provided feedback. Throughout the analysis, reflexive memos were maintained to document analytic decisions and reduce potential researcher bias. These steps helped strengthen the dependability and confirmability of the qualitative findings, which served to complement and contextualize the quantitative results.

### Ethical Considerations

This study was approved by the institutional review board of the principal investigator’s institution (institutional review board number ewha-202407-0025-01) and registered in the Clinical Research Information Service (KCT0009853; October 16, 2024). All participants received detailed information about the study’s purpose, procedures, risks, and benefits through an online study information page, and electronic informed consent was obtained prior to enrollment. Participants were informed of their right to withdraw from the study at any time without penalty. To ensure privacy and confidentiality, all collected data were deidentified and stored in encrypted, password-protected files. Contact information used for randomization and compensation delivery was stored separately from outcome data, and only authorized research personnel had access to the deidentified dataset. All participants received a token of appreciation valued at KRW 5000 (approximately US $4) for each survey completed (baseline, posttest 1, and posttest 2). In addition, experimental group participants who took part in the optional follow-up interview received an additional KRW 20,000 (approximately US $15) gift card as compensation.

## Results

### Homogeneity of Demographic Characteristics and Study Variables Between Groups

**Figure 1.**
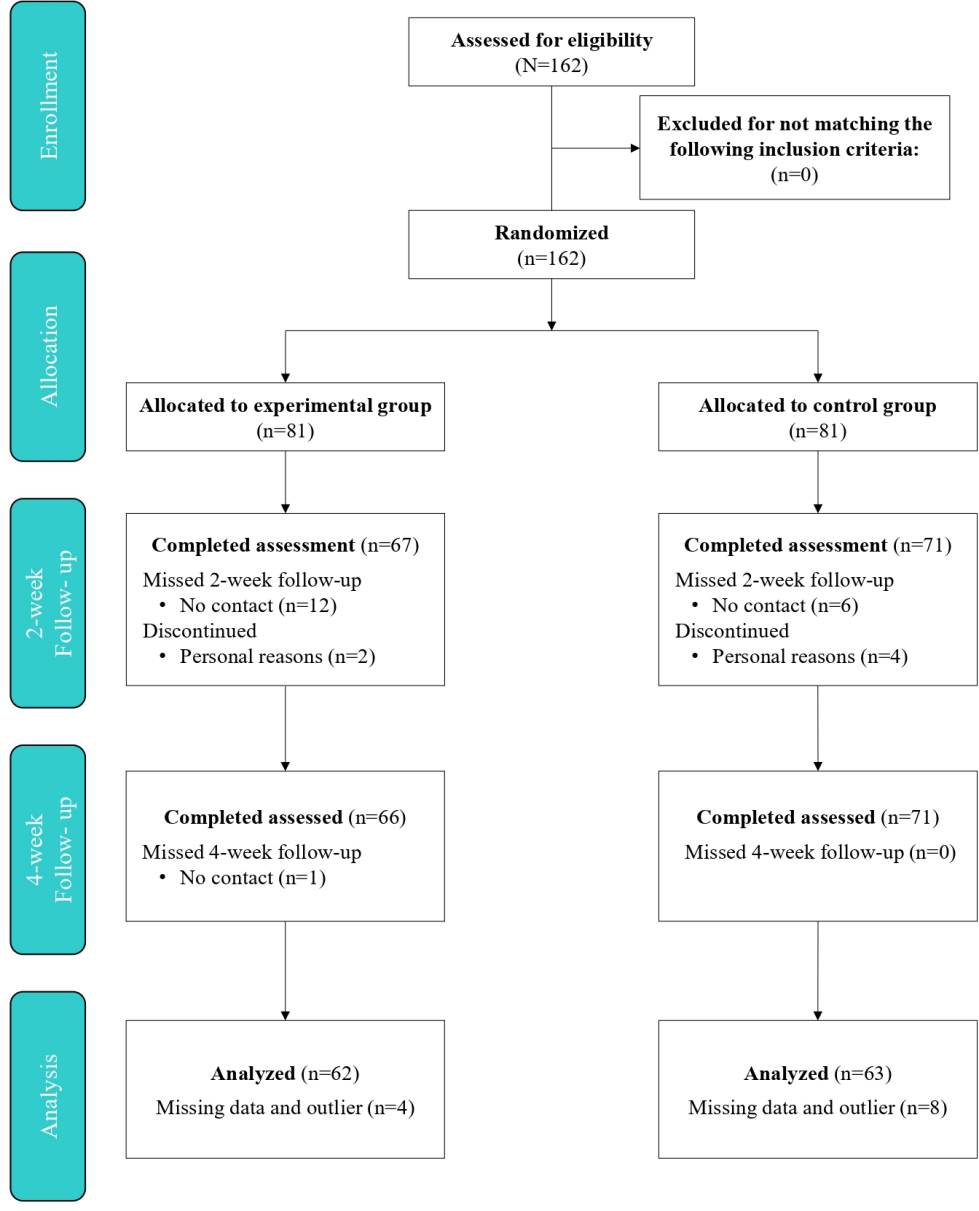
CONSORT (Consolidated Standards of Reporting Trials) diagram profile flow.

Baseline comparisons indicated no significant differences in demographic characteristics between groups (Table 2). However, job stress (*t*_123_=3.80, *P*<.001) and stress response (*t*_123_=3.77, *P*<.001) differed significantly and were therefore included as covariates in subsequent analyses to adjust for potential confounding effects (Figure 1).

**Table 2.** Homogeneity of demographic and study variables between the groups (N=125).

Variables and categories	Exp.^a^ (n=62)	Cont.^b^ (n=63)	Chi-square (*df*) or *t* (*df*)	*P* value
Age (y), mean (SD)	28.18 (5.49)	27.81 (3.76)	0.44 (124)	.66
Age (y), n (%)			5.36 (1)	.15
<30	43 (69.4)	41 (65.1)		
≥30	19 (30.6)	22 (34.9)		
Sex, n (%)			1.31 (1)	.44
Female	60 (96.8)	58 (92.1)		
Male	2 (3.2)	5 (7.9)		
Marital status, n (%)			3.01 (1)	.22
Single	54 (87.1)	58 (92.1)		
Married	8 (12.9)	5 (7.9)		
Job position, n (%)			1.53 (1)	.47
Staff nurse	55 (88.7)	59 (93.7)		
Charge/head nurse	7 (11.3)	4 (6.3)		
Hospital size (beds), n (%)			9.29 (2)	.06
≤100	7 (11.3)	9 (14.2)		
101-500	36 (58.1)	26 (41.4)		
≥500	19 (30.6)	28 (44.4)		
Clinical experience (y), mean (SD)	4.21 (4.43)	3.71 (3.03)	0.73 (124)	.47
Clinical experience (y), n (%)			1.50 (1)	.22
≤5	40 (64.5)	47 (74.6)		
>5	22 (35.5)	16 (25.4)		
Working department, n (%)			7.26 (2)	.30
General ward	43 (69.4)	37 (58.7)		
ICU^c^/ER^d^	14 (22.6)	17 (27.0)		
Others^e^	5 (8.0)	9 (14.3)		
Overtime on average during the past month (h), n (%)			1.35 (4)	.93
≤0.5	29 (46.7)	30 (47.6)		
≤1	17 (27.4)	19 (30.2)		
≤1.5	8 (12.9)	5 (7.9)		
≤2	4 (6.5)	5 (7.9)		
>2	4 (6.5)	4 (6.3)		
Type of shift, n (%)			5.83 (2)	.12
8-h shift	53 (85.5)	49 (77.8)		
12-h shift	0 (0.0)	1 (1.6)		
Fixed	9 (14.5)	13 (20.6)		
Turnover intention, mean (SD)	5.61 (2.29)	5.37 (2.29)	0.61 (124)	.55
Burnout, mean (SD)				
Total score	63.01 (17.57)	61.49 (18.54)	0.47 (124)	.64
Client-related burnout	59.88 (24.17)	53.90 (22.44)	1.43 (124)	.15
Personal burnout	71.10 (18.57)	66.73 (19.72)	1.28 (124)	.21
Work-related burnout	58.76 (16.56)	63.49 (19.25)	−1.47 (124)	.14
Coping strategy, mean (SD)	2.08 (0.28)	2.10 (0.26)	−0.35 (124)	.72
Job stress, mean (SD)	3.95 (0.44)	3.65 (0.44)	3.80 (124)	<.001
Stress response, mean (SD)	2.05 (0.66)	1.55 (0.80)	3.77 (124)	<.001

a Exp.: experimental group.

b Cont.: control group.

c ICU: intensive care unit.

d ER: emergency room.

e Others: operating room, recovery room, delivery room, outpatient, administration.

### Effects of the Intervention on Nurse Burnout

**Figure 2.**
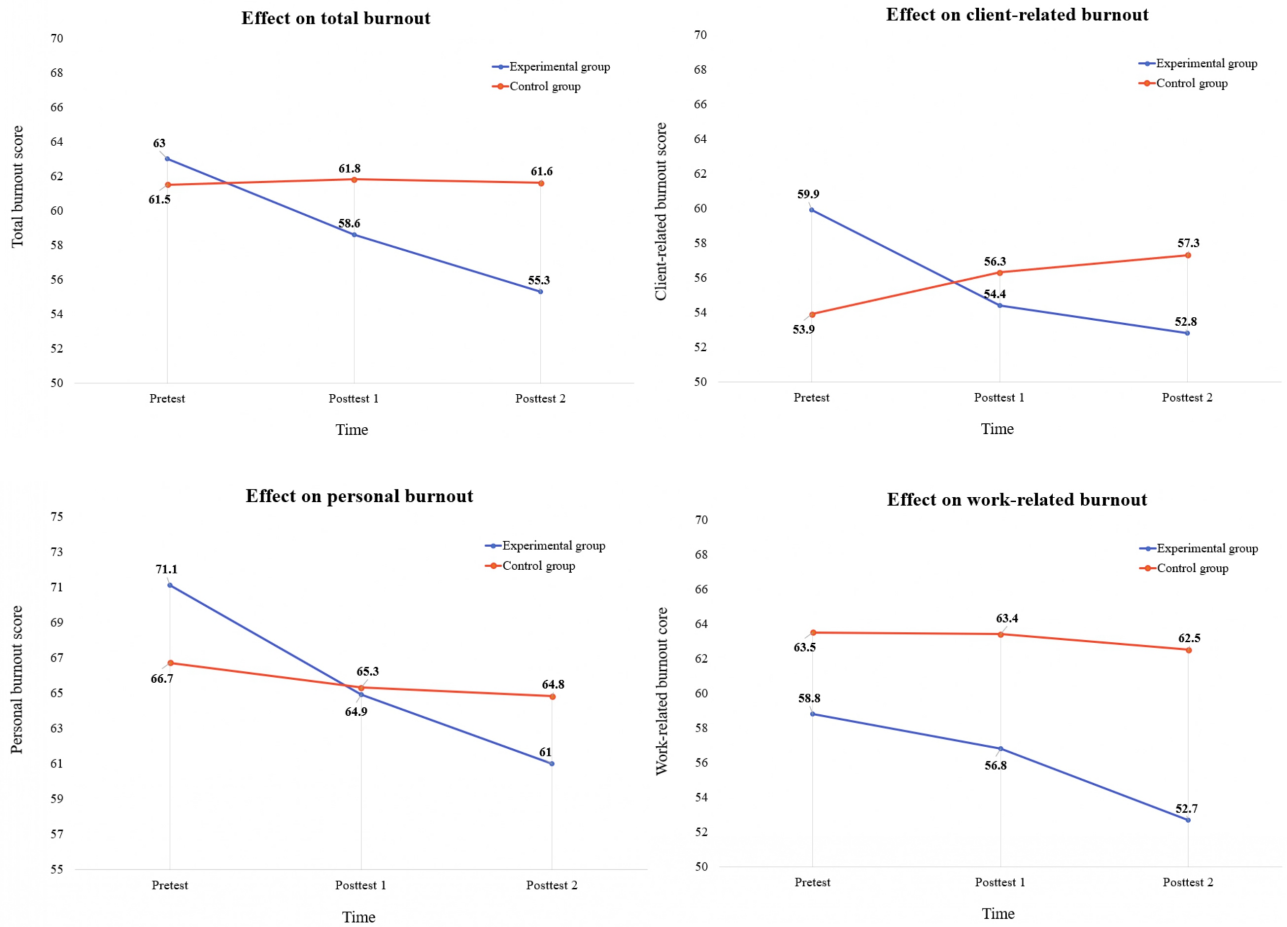
Trends in burnout scores over time, based on repeated-measures ANOVA with covariates.

Figure 2 and Table 3 present the repeated-measures ANOVA results with covariates. The experimental group showed significantly greater reductions than the control group in client-related burnout (*F*_1,121_=7.548, *P*=.007), personal burnout (*F*_1,121_=6.533, *P*=.01), and work-related burnout (*F*_1,121_=38.194, *P*<.001), reflecting significant group effects. No significant time or interaction effects were observed. In addition, paired-sample *t* tests indicated significant within-group reductions from baseline to posttest 2 in the experimental group across total burnout and all burnout subdomains, with small-to-moderate effect sizes. However, no significant within-group changes were observed in the control group.

**Table 3.** Paired-sample *t* tests and repeated-measures ANOVA with covariates (N=125).

Study variables and group	Pretest, mean (SD)	Posttest 1, mean (SD)	Posttest 2, mean (SD)	Difference^a^, mean (SD)	Paired *t* test (*P* value)	Cohen *d*	Repeated-measures ANOVA, *F* (*df*=1,121) (*P* value)
Total burnout score							2.943 (.06)^b^
Exp.^c^ (n=62)	63.01 (17.57)	58.62 (17.25)	55.30 (23.11)	7.70 (19.82)	3.06 (.003)	0.39	16.960 (<.001)^d^
Cont.^e^ (n=63)	61.49 (18.54)	61.77 (18.31)	61.59 (21.60)	−0.10 (14.13)	−0.06 (.95)	−0.01	0.718 (.47)^f^
Client-related burnout							2.923 (.06)^b^
Exp. (n=62)	59.88 (24.17)	54.44 (21.99)	52.76 (25.02)	7.64 (24.93)	2.34 (.02)	0.30	7.548 (.007)^d^
Cont. (n=63)	53.90 (22.44)	56.28 (22.30)	57.34 (24.10)	−4.05 (19.13)	−1.40 (.17)	−0.18	0.384 (.66)^f^
Personal burnout							2.596 (.08)^b^
Exp. (n=62)	71.10 (18.57)	64.92 (19.39)	60.95 (26.87)	10.15 (20.67)	3.87 (<.001)	0.49	6.533 (.01)^d^
Cont. (n=63)	66.73 (19.72)	65.34 (19.05)	64.81 (23.24)	1.92 (15.77)	0.97 (.34)	0.12	0.283 (.74)^f^
Work-related burnout							1.669 (.19)^b^
Exp. (n=62)	58.76 (16.56)	56.80 (15.00)	52.65 (21.69)	6.11 (20.90)	2.30 (.03)	0.29	38.194 (<.001)^d^
Cont. (n=63)	63.49 (19.25)	63.44 (19.48)	62.47 (21.62)	1.02 (14.21)	0.57 (.57)	0.07	1.854 (.16)^f^

a Differences: pretest score—posttest 2 score.

b Source: Interaction effects.

c Exp.: experimental group.

d Source: Group effects.

e Cont.: control group.

f Source: Time effects.

### Effects of the Intervention on Coping Strategy, Job Stress, and Stress Response

Table 4 presents between-group comparisons of coping strategy, job stress, and stress response from pretest to posttest 2. No statistically significant between-group differences were observed for these secondary outcomes. Although the experimental group showed numerically greater reductions in job stress and stress response compared with the control group, the between-group differences effect sizes were small to modest in magnitude.

**Table 4.** Independent *t* test: group differences in job stress, stress response, and coping strategy (N=125).

Study variables and group	Pretest, mean (SD)	Posttest 2, mean (SD)	Differences^a^, mean (SD)	*t* (*df*=123) (*P* value)	Cohen *d*
Coping strategy				−1.358 (.18)	−0.24
Exp.^b^ (n=62)	2.08 (0.28)	2.15 (0.35)	−0.07 (0.35)		
Cont.^c^ (n=63)	2.10 (0.26)	2.09 (0.33)	0.01 (0.31)		
Job stress				1.848 (.07)	0.33
Exp. (n=62)	3.95 (0.44)	3.64 (0.81)	0.31 (0.66)		
Cont. (n=63)	3.65 (0.44)	3.56 (0.67)	0.09 (0.66)		
Stress response				1.237 (.22)	0.22
Exp. (n=62)	2.05 (0.66)	1.77 (0.83)	0.28 (0.79)		
Cont. (n=63)	1.55 (0.80)	1.44 (0.88)	0.11 (0.76)		

a Differences: pretest score—posttest 2 score.

b Exp.: experimental group.

c Cont.: control group.

### Participant Experiences With the Intervention

#### Overview

We identified 2 overarching themes: experiences with the AI algorithm and perceptions of the burnout reduction program. Subthemes within each theme reflected participants’ emotional, cognitive, and behavioral responses during the intervention.

#### Experiences With the Burnout Reduction Program

Participants described the program as an opportunity for self-reflection, emotional relief, and the development of coping strategies applicable to daily life.

##### Reflecting on Oneself

Several participants noted that guided activities, such as reflective writing and mindfulness meditation, enabled them to reassess their emotional state and recognize personal growth. These activities facilitated intentional self-observation and reflection on past experiences. For example, 1 participant explained:

*During reflective writing, I looked back on past memories. It was tough then, but now I could see how much I have grown*.

Another participant stated:

*The meditation gave me space to empty my mind and reflect. Having time just for myself was the biggest change*.

##### Feeling Calmer Overall

Participants reported feeling more relaxed and emotionally composed through the program’s cognitive and physical relaxation components, which, in some cases, carried over into their clinical practice. One participant noted:

*I do not know if it helped with burnout directly, but I felt more at ease. I felt calmer*.

Another participant shared:

*When dealing with patients, I responded more gently instead of reacting harshly. I could keep calm even in busy environments*.

##### Creating a Mental Space for Recovery

Participants described how emotional residue from clinical work often lingered after shifts, exacerbating burnout. The program provided temporary psychological detachment from work-related stressors and created a mental space for recovery. One participant explained:

*I used to just eat or sleep after work, but even after sleeping, the stress did not go away. This program helped me forget about it for a while*.

Another participant noted:

*Focusing on the video and sound helped me forget things, even if just briefly*.

##### Strengthening Self-Efficacy Through Personalized Coping

Participants reported increased confidence in managing stress and preventing burnout. By identifying coping techniques suited to their needs and integrating them into daily life, they strengthened their sense of self-efficacy. One participant shared:

*Stretching helped me regulate my emotions, and now I stretch as a daily habit*.

Another participant explained:

*When I am struggling, I try taking deep breaths or moving my body—my own way of coping*.

### Experiences With the AI Algorithm

Participants were often introduced to interventions they had not previously considered. They also expressed a strong sense of trust in the AI-based recommendation process.

#### Exposure to New Programs Beyond Preexisting Preferences

Participants noted that the AI algorithm encouraged them to engage in programs they would not have selected independently, thereby reducing reliance on habitual or preference-driven choices. One participant reflected:

*As people, we tend to choose what we already know. But because the AI recommended something different, I could experience a variety of options*.

Another participant added:

*It was a refreshing experience. I could try methods I would not have chosen myself, and they turned out to work surprisingly well for me*.

#### Trust in AI-Based Recommendations

Participants expressed confidence in the AI-generated recommendations, emphasizing that the suggestions felt objective and tailored to their personal condition. These comments suggest that some participants were receptive to the AI-recommended programs, even when the content differed from what they would have personally preferred. One participant noted:

*When friends recommend something, I sometimes dismiss it because they do not really know me. However, with AI, it feels like it is based on a thorough understanding of my condition*.

Another participant added:

*If I chose a program myself, I would have picked something I liked, not necessarily what I needed. The AI seemed to know what was appropriate for me at the moment*.

## Discussion

### Principal Findings

In this study, a greater reduction was observed in all 3 burnout subdomains in the experimental group compared with the control group. However, these results should be interpreted in light of the absence of significant time and group interaction effects. These findings suggest a potential role for tailored interventions in supporting burnout reduction, although confirmatory evidence demonstrating significant interaction effects is still needed. Previous studies have suggested the role of CBT in mitigating burnout [15,16] and extending it by integrating AI-based personalization into mHealth delivery. Previous studies applied standardized programs to all participants without accounting for individual differences in burnout profiles or coping strategies [38,39]. A recent meta-analysis also confirmed that mobile CBT-based interventions generally reduce stress and burnout among nurses but noted heterogeneity in effects due to intervention type and user engagement [40]. This study assists in addressing these gaps by using an AI algorithm that tailors CBT-based interventions to users’ demographic, work-related characteristics, and psychological profiles.

Although several mHealth interventions, such as Wysa and Woebot, have demonstrated effectiveness in reducing psychological symptoms through chatbot-delivered CBT, these platforms typically tailor content based on brief, momentary self-reported mood or symptom check-ins [19,20]. By contrast, this study used a data-driven recommendation algorithm informed by multidimensional baseline self-report data, including burnout subdomains, job stress levels, and coping styles, to guide the selection of CBT-based modules. This approach was intended to support a relatively more condition-specific and theoretically grounded personalization strategy rather than real-time mood-based content adaptation. The theoretical grounding of this approach is based on a multidimensional conceptualization of burnout, which assumes that different burnout subtypes are associated with distinct cognitive and behavioral mechanisms [7]. Accordingly, the recommendation algorithm was designed to align users’ burnout profiles and coping characteristics with CBT-based modules targeting specific therapeutic mechanisms, such as cognitive restructuring, behavioral activation, and emotion regulation [15,16]. Moreover, while many digital mental health interventions primarily rely on quantitative outcome metrics, this study additionally incorporated a qualitative component to capture participants’ lived experiences with the AI-assisted program. The thematic analysis highlighted users’ reflections on increased self-awareness [41], trust in AI recommendations, and emotional relief, thereby providing contextual insight into the observed quantitative results.

Despite the generally favorable trends observed, the magnitude of the observed changes in burnout was modest, which necessitates a cautious interpretation of the direct effectiveness of the AI-based mobile CBT-based intervention. This pattern is consistent with findings from a recent systematic review of person-directed psychoeducational interventions for nurses, which reported that CBT-based approaches generally contribute to reductions in burnout while also demonstrating considerable variability in effect sizes and limited evidence regarding the long-term sustainability of effects [42]. Furthermore, the fully remote and self-guided nature of the intervention may have contributed to variability in how participants engaged with the program, which could partially account for the modest intervention effects observed in this study. Future studies should incorporate more comprehensive adherence-reporting methods, including objective indicators of behavioral engagement, such as module completion rates, frequency of session participation, usage duration, and interaction logs. Such approaches may help clarify the relationship between intervention engagement and treatment outcomes in fully remote CBT-based interventions. Despite these limitations, the qualitative results of this study provide important contextual insight into how such interventions may exert their influence. Rather than indicating immediate or substantial symptom reduction, participants described gradual changes in self-awareness, coping orientation, and engagement with stress-related situations. These experiential changes suggest that AI-selected mobile CBT-based interventions may operate by facilitating early-stage cognitive and behavioral adjustments, which may precede more pronounced reductions in burnout over time. Integrating these qualitative insights with the quantitative outcomes allows for a more nuanced interpretation of the intervention effects, particularly in the context of digital and mobile-based burnout interventions for nurses.

Although mobile and AI-assisted interventions are often assumed to reduce dropout by improving accessibility and flexibility, the attrition rate in this study approached 20% [11], which is comparable to rates reported in face-to-face CBT-based interventions. This finding suggests that the mobile format alone may not be sufficient to substantially reduce dropout among nurses. Factors such as high baseline occupational stress, shift work, and limited cognitive and emotional resources may have constrained sustained engagement, even in a self-guided and flexible intervention context [2]. These findings indicate that although mobile delivery may lower structural barriers, additional strategies, such as adaptive reminders, enhanced human support, or shorter intervention modules, may be necessary to further improve retention in future digital burnout interventions for nurses.

A particularly noteworthy finding of this study is that participants reported engaging in coping strategies they might not have selected on their own, specifically because they were recommended by the AI algorithm. From the interviews, participants reported that AI-generated recommendations encouraged engagement with unfamiliar coping strategies that were later perceived to be helpful. Such accounts indicate that the AI recommendations may have contributed to prompting participants to move beyond their habitual coping tendencies and consider alternative strategies. Prior research suggests that individuals experiencing burnout tend to exhibit reduced cognitive flexibility due to emotional exhaustion, often resorting to avoidant and passive coping strategies, such as denial, self-blame, and behavioral disengagement [43,44]. Although these strategies may temporarily conserve psychological energy, they ultimately fail to address the root causes of stress and can perpetuate a cycle of worsening burnout. Within this context, our findings suggest that the AI-selected intervention did more than merely provide additional options. By tailoring recommendations to participants’ burnout subdomains and psychological profiles, it may have helped reduce preference-driven selection and encouraged consideration of strategies that participants might otherwise have overlooked. In this sense, the AI algorithm functioned not only as a personalization tool but also as a facilitator that broadened participants’ coping repertoires and supported openness to new and potentially more adaptive approaches.

Moreover, the participants who were interviewed expressed a high level of trust in the AI-generated recommendations. This trust appeared to stem from their perception that the recommendations were objective and data-driven; however, this interpretation should be viewed cautiously, as the study did not include a comparison condition involving recommendations from human professionals. Prior research has shown that some individuals tend to attribute greater objectivity to algorithmic guidance [45], and trust is known to be a key factor influencing engagement with and continued use of digital mental health interventions [46]. In this study, participants’ trust in the AI-assisted program may have supported openness toward continued participation, although the relative advantage of AI-generated recommendations over human-delivered recommendations cannot be inferred from the current study design. Furthermore, this study suggests that AI-based mental health interventions may represent a promising complementary approach to conventional group-based or offline burnout programs, which often face structural limitations. Traditional interventions rely on fixed formats, predetermined schedules, and in-person settings, making them less accessible or feasible for professionals with demanding schedules, such as shift-working nurses. A systematic review reported that many burnout interventions were group-based and conducted offline, and they consistently noted barriers, such as scheduling difficulties, participant dropout, and lack of long-term effectiveness [42]. By contrast, the AI algorithm applied in this study delivered tailored CBT-based modules by automatically analyzing multidimensional user data, including burnout subtypes, stress levels, and coping styles. This approach enabled precise and flexible intervention without time or location constraints.

Such technological adaptability is not limited to specific professions but offers the scalability and inclusiveness needed to support high-risk occupational groups (eg, teachers, firefighters, and social workers) as well as vulnerable populations (eg, adolescents, older adults, and informal caregivers). As data from more diverse groups accumulate and algorithmic learning advances, AI interventions may become increasingly capable of addressing a broader range of mental health conditions, such as anxiety, depression, sleep disorders, and posttraumatic stress disorder. In this regard, AI-powered digital interventions may offer a scalable and sustainable approach that simultaneously enhances accessibility and personalization—2 essential elements in the future of mental health services.

### Limitations

This study has several limitations that should be considered when interpreting the findings. First, all outcome measures were self-reported, thereby raising concerns about potential reporting bias, including social desirability. Second, participants were recruited without a minimum screening threshold for burnout symptoms. Although the mean baseline burnout scores exceeded 50, indicating generally elevated burnout levels among participants, the absence of a formal screening process means that participants were not explicitly selected based on a confirmed need for intervention. Although this design allows for the examination of the AI-selected mobile CBT-based intervention in real-world nurses who are vulnerable to burnout, it limits the ability to draw conclusions about intervention effects among individuals with suspected or clinically significant burnout. Future studies should consider targeting participants with elevated burnout levels to more directly test intervention efficacy. Third, although participants’ access to the intervention platform and participation status were monitored digitally, this study did not include objective adherence indicators reflecting the depth of engagement with the intervention, such as total usage duration, module completion rates, session completion frequency, or detailed interaction logs. As a result, it was not possible to determine the extent to which participants actively engaged with the intervention content throughout the study period. Because this study used a fully remote, self-guided intervention format, the absence of more robust adherence data limits the interpretation of the observed intervention effects and may partially explain the relatively modest or inconsistent effects observed across several outcomes. Future studies should incorporate more comprehensive adherence-reporting methods, including objective behavioral engagement indicators, to better evaluate the relationship between intervention engagement and treatment outcomes. Fourth, although this study was conducted as a single-blind randomized controlled trial, complete participant blinding could not be fully guaranteed. Because the control group received only informational materials while the experimental group engaged with interactive AI-selected modules, participants may have inferred their group assignment. Such potential unblinding may have influenced their expectations or engagement levels, thereby introducing performance bias. Fifth, this study did not include a comparison group receiving recommendations from human professionals. As a result, interpretations regarding the relative superiority or added benefit of AI-generated recommendations should be made with caution. Sixth, baseline levels of job stress and stress response were significantly higher in the experimental group than in the control group, and these variables were statistically adjusted for using analysis of covariance. However, such baseline heterogeneity may have influenced responsiveness to the intervention, thereby complicating interpretation of the observed group differences. Participants with higher baseline psychological distress may have responded differently to the intervention compared with less distressed participants. Future studies should consider stratified randomization or subgroup analyses to better account for baseline psychological differences. Seventh, this study did not use an ITT analysis. Excluding participants with missing data or outliers may have reduced the benefits of randomization and introduced potential selection bias. Future studies should consider applying ITT approaches, such as mixed-effects modeling or multiple imputation, to better preserve randomization and enhance the robustness of the findings.

### Conclusions

This study evaluated the effectiveness of an AI-selected, mobile, CBT-based intervention in reducing nurse burnout and explored participants’ experiences with how the AI-selected program supported them during the intervention period. By tailoring program selection based on individual characteristics and burnout-related factors, the intervention was designed to address some limitations of standardized approaches. Nurses who received the AI-selected intervention showed reductions in burnout, suggesting the potential use of personalized digital mental health interventions as a supportive approach for nurse burnout management. These findings highlight the potential of AI technology to enhance individualized care in occupational mental health and support further studies to examine its long-term effects and applicability across diverse health care settings.

## Supplementary material

10.2196/85986 Checklist 1 CONSORT checklist.

## Abbreviations

AI: artificial intelligence
CBT: cognitive behavioral therapy
CONSORT: Consolidated Standards of Reporting Trials
COREQ: Consolidated Criteria for Reporting Qualitative Research
ITT: intention-to-treat
mHealth: mobile health
